# Exploring the Molecular Mechanism of 1,25(OH)_2_D_3_ Reversal of Sorafenib Resistance in Hepatocellular Carcinoma Based on Network Pharmacology and Experimental Validation

**DOI:** 10.3390/cimb47050319

**Published:** 2025-04-29

**Authors:** Zhiyan Long, Xiangyi Wu, Tianxin Luo, Xiaomei Chen, Jian Huang, Shu Zhang

**Affiliations:** 1 Center for Clinical Laboratories, The Affiliated Hospital of Guizhou Medical University, Guiyang 550001, China; 2022110050988@stu.gmc.edu.cn (Z.L.); 2022110050995@stu.gmc.edu.cn (X.W.); 18798038754@163.com (T.L.); 18798872075@163.com (X.C.); 2 Department of Basic Clinical Laboratory Medicine, School of Clinical Laboratory Science, Guizhou Medical University, Guiyang 550001, China

**Keywords:** 1,25(OH)_2_D_3_, hepatocellular carcinoma sorafenib resistance, chemosensitivity, network pharmacology, autophagy, FOXO3A/FOXM1 signaling axis

## Abstract

Sorafenib is currently the first-line therapeutic agent for advanced hepatocellular carcinoma (HCC). However, sorafenib resistance remains a major clinical challenge. Studies have reported that 1,25-dihydroxyvitamin D3 (1,25(OH)_2_D_3_) can synergize with multiple chemotherapeutic drugs to enhance their antitumor efficacy, but the combinatorial effect between 1,25(OH)_2_D_3_ and sorafenib has not yet been investigated. This study aimed to investigate the potential molecular mechanism by which 1,25(OH)_2_D_3_ reverses sorafenib resistance in hepatocellular carcinoma using network pharmacology, molecular docking, and experimental validation. We predicted a web-based pharmacological approach to predict potential targets of 1,25(OH)_2_D_3_ and its derivatives, as well as sorafenib resistance genes in hepatocellular carcinoma from public databases. We then constructed 1,25(OH)_2_D_3_ chemo-sensitizing expression profiles through intersection analysis. Gene Ontology (GO) and Kyoto Encyclopedia of Genes and Genomes (KEGG) pathway enrichment analyses were employed to predict the potential pathways involved in 1,25(OH)_2_D_3_ chemosensitization, followed by molecular docking analysis and analysis of molecular dynamics simulations. Finally, experimental validation were conducted to elucidate the potential mechanisms by which 1,25(OH)_2_D_3_ enhances the sensitivity of HCC to sorafenib. Compound and target screening identified 730 predicted targets of 1,25(OH)_2_D_3_ and its derivatives, 1144 genes associated with sorafenib resistance in hepatocellular carcinoma, and 56 potential chemosensitization targets from the intersection analysis. KEGG analysis suggested that the chemosensitization effect of 1,25(OH)_2_D_3_ might be mediated by the FoxO signaling pathway. Molecular docking showed that both 1,25(OH)_2_D_3_ and its derivatives could stably bind to FOXO3A, a key gene in the FoxO family, and molecular dynamics simulation analysis further indicated that the two bind well together. In vitro experiments demonstrated the synergistic effects of 1,25(OH)_2_D_3_ and sorafenib, significantly inhibiting the viability and colony formation rate of sorafenib-resistant hepatocellular carcinoma cells. Additionally, the combination treatment promoted apoptosis and inhibited autophagy. Furthermore, the combination modulated the FOXO3A/FOXM1 signaling axis. This study reveals that 1,25(OH)_2_D_3_ enhances the chemosensitivity of hepatocellular carcinoma (HCC) to sorafenib, with underlying mechanisms potentially involving the targeted modulation of the FOXO3A/FOXM1 signaling axis and the reversal of sorafenib-resistant phenotypes through the regulation of apoptotic and autophagic pathways.

## 1. Introduction

Hepatocellular carcinoma (HCC) is one of the most common malignant tumors in the world, with a high mortality rate, posing a serious threat to human health globally [1]. Because the symptoms and signs of early HCC are atypical and difficult to detect, more than 50% of HCC patients are diagnosed at the middle or late stages of the disease [1,2]. Sorafenib is a multi-targeted tyrosine kinase inhibitor that promotes apoptosis, attenuates angiogenesis, and inhibits tumor cell proliferation, potentially improving overall survival in patients with intermediate and advanced HCC [3]. However, liver cancer cells can develop resistance to sorafenib through various mechanisms, resulting in limited long-term benefits. Typically, patients become resistant to sorafenib treatment within six months [4].

1,25-dihydroxyvitamin D3 (1,25(OH)_2_D_3_, calcitriol) is the active form of vitamin D3, widely recognized for its role in maintaining calcium and phosphate homeostasis [5]. In addition to its classical effects on bones, kidneys, and the intestinal tract, 1,25(OH)_2_D_3_ and its analogs have been extensively studied for their antitumor effects on various types of cancer [6,7]. Research conducted in the United States has shown that 1,25(OH)_2_D_3_ and its analogs can enhance the inhibitory effects of drugs in cancer treatment when used in combination with other agents [8]. For instance, Ma et al. demonstrated that 1,25(OH)_2_D_3_ sensitizes squamous cell carcinoma cells to cisplatin-induced cytotoxicity and apoptosis using both in vivo and in vitro models [9]. Hershberger et al., in vitro, showed that pretreatment with 1,25(OH)_2_D_3_ enhances the growth-inhibitory effects of paclitaxe [10]. Additionally, independent studies have reported that combining 1,25(OH)_2_D_3_ with various tyrosine kinase inhibitors (TKIs) such as gefitinib/erlotinib (EGFR inhibitors) and sunitinib (a multi-targeted TKI) induces the differentiation of AML cells [11]. Our previous studies have demonstrated that 1,25(OH)_2_D_3_ inhibits the proliferation of hepatocellular carcinoma cells. However, no research has yet reported the relationship between 1,25(OH)_2_D_3_ and sorafenib resistance in liver cancer. Therefore, this study combined 1,25(OH)_2_D_3_ with sorafenib to investigate whether 1,25(OH)_2_D_3_ could enhance the chemosensitivity of sorafenib in hepatocellular carcinoma treatment.

Network pharmacology allows for the exploration of the mechanisms by which compounds treat diseases [12]. Integrated network pharmacology approaches combined with Kyoto Encyclopedia of Genes and Genomes (KEGG) pathway analysis and bioinformatics interrogation demonstrated that 1,25(OH)_2_D_3_ may potentiate sorafenib chemosensitivity in HCC through the modulation of the FoxO signaling pathway, with FOXO3A identified as a pivotal regulatory mediator, a key protein in the FoxO signaling pathway and a member of the Forkhead family of transcription factors; it plays a crucial role in liver cancer, breast cancer, colon cancer, and other cancers [13]. Located in the nucleus, FOXO3A actively mediates various cellular processes such as apoptosis induction through the transcription of its target genes in their non-phosphorylated form [14], as well as processes related to proliferation [15] and the cell cycle [16]. In addition, FOXO3A plays a crucial role in various aspects of cancer progression and drug resistance. Recent studies have indicated that dysregulation of FOXO3A promotes drug resistance through multiple mechanisms, including the regulation of oncogenic signaling pathways, evasion of apoptosis, and induction of autophagy. Therefore, we hypothesize that FOXO3A dysregulation may contribute to sorafenib resistance in hepatocellular carcinoma by influencing apoptosis and autophagy processes.

An integrated methodology combining network pharmacology, molecular docking, and in vitro experimental validation was implemented to preliminarily investigate the molecular targets and mechanistic basis underlying the 1,25(OH)_2_D_3_-mediated enhancement of sorafenib chemosensitivity in hepatocellular carcinoma.

## 2. Materials and Methods

### 2.1. GEO Microarray and Acquisition of Sorafenib-Resistant Targets in Hepatocellular Carcinoma

The Gene Expression Omnibus (GEO) database, curated by the National Center for Biotechnology Information (NCBI), serves as a comprehensive repository for high-throughput omics data, housing extensive datasets pertinent to tumor drug resistance mechanisms. The keyword “liver cancer sorafenib resistance” was utilized in the GEO database [17]. The dataset GSE94550 was downloaded from http://www.ncbi.nlm.nih.gov/geo/ (accessed on 20 January 2024) and analyzed using GEO2R to identify differentially expressed genes. A *p*-value threshold of less than 0.05 and |logFC| greater than 1 were employed as criteria for the up- and down-regulation of the identified genes. The results of differentially expressed genes were visualized through the Xiantao Online website and presented in volcano plots.

### 2.2. Target Prediction for 1,25(OH)_2_D_3_ and Its Derivatives

1,25(OH)_2_D_3_ and its derivatives were obtained in SMILE format from the PubChem database [18] (https://pubchem.ncbi.nlm.nih.gov (accessed on 20 January 2024). Swiss Target Prediction [19] was used to predict targets for 1,25(OH)_2_D_3_ and its derivatives. Targets with a prediction probability > 0.1 in the Swiss Target Prediction Database were selected, and duplicates were removed to obtain the final targets.

### 2.3. Prediction of Potential Targets for 1,25(OH)_2_D_3_ Chemosensitization

Venn analysis was performed using the Xanadu Online website to analyze the predicted targets of 1,25(OH)_2_D_3_ and its derivatives along with genes associated with sorafenib resistance in hepatocellular carcinoma. The intersection of these sets was considered a potential target for reversing sorafenib resistance by 1,25(OH)_2_D_3_ and its derivatives.

### 2.4. Biofunctional Analysis

The identified potential targets were analyzed through Gene Ontology (GO) and Kyoto Encyclopedia of Genes and Genomes (KEGG) using the DAVID database (https://david.ncifcrf.gov/ (accessed on 21 January 2024), an online bioinformatics platform. This analysis aimed to acquire comprehensive functional annotation details for the targets. Annotations were considered to be significantly enriched when they met a significance threshold of *p* < 0.05. To present the enrichment results in a more intuitive way, the bioinformatics online analysis website https://www.bioinformatics.com.cn (accessed on 21 January 2024) was employed for visualization.

### 2.5. Construction of “Compound–Target” and “Compound–Target–Pathway” Network Diagrams

The data, comprising 1,25(OH)_2_D_3_, its derivatives, predicted targets, and enriched pathways, were imported into Cytoscape v3.7.2 for network visualization. We constructed two interactive networks: a compound–target interaction network and a compound–target–pathway association network. In the graphical representation, each constituent, target gene and pathway was described by node, and the interactions were encoded by edges.

### 2.6. Molecular Docking

The 3D structure of FOXO3A was retrieved from the PDB database https://www.rcsb.org/ (accessed on 2 March 2024), and the target proteins were pre-treated by desolvation and the removal of small organic molecules using PyMOL software. The 2D structures of 1,25(OH)_2_D_3_ and sorafenib were obtained from the PubChem database https://pubchem.ncbi.nlm.nih.gov/ (accessed on 2 March 2024), and these small-molecule structures were converted into 3D structures with energy minimization using Chem3D Ultra 14.0 software. AutoDock 4.2.6 software [20] was employed to add hydrogen atoms to both proteins and small molecules, and molecular docking was conducted after setting rotatable bonds for the small molecules. Binding free energy (ΔG) serves as the central quantitative metric for evaluating the stability of ligand–receptor complexes, a process where more negative values correlate with stronger binding affinity and enhanced complex stabilization [21]. A binding energy ≤ −7 kcal/mol [22,23,24] was generally used as a screening criterion to evaluate the binding stability of FOXO3A with 1,25(OH)_2_D_3_ and sorafenib, and the lowest binding energy model was selected for the visualization and analysis by PyMOL 2.4.0 software.

### 2.7. Molecular Dynamics Simulations

We performed 100 ns MD simulations of the complexes using Gromacs 2023 software. The CHARMM 36 [25] force field parameters were used for the protein and the ligand topology was constructed from the GAFF2 force field parameters. Periodic boundary conditions were used and the protein–ligand complexes were placed in cubic boxes. The TIP3P water model [26] was used to fill the box with water molecules. Electrostatic interactions were treated using the Particle Mesh Ewald (PME) and Verlet algorithms, respectively. Subsequently, 100,000 steps of isothermal isovolumic systematic equilibrium and isothermal isobaric systematic equilibrium were performed with a coupling constant of 0.1 ps and a duration of 100 ps simulation. Both van der Waals and Coulomb interactions were calculated using a cut-off value of 1.0 nm. Finally, the system was subjected to molecular dynamics simulations using Gromacs 2023 at constant temperature (300 K) and constant pressure (1 bar) for a total duration of 100 ns.

### 2.8. Cell Culture

Hepatocellular carcinoma parental cells, Huh7, and sorafenib-resistant hepatocellular carcinoma cells, Huh7/s, were obtained from the Cell Bank Type Culture Preservation Centre of OLU Bioscience and Technology Co., Ltd. (Shanghai, China). The cells were cultured in RPMI-1640 medium supplemented with 10% fetal bovine serum and 1% penicillin–streptomycin solution (100×). They were incubated under standard conditions (37 °C, 5% CO_2_, 95% humidity), and experiments were conducted when the cells reached 80–90% confluence. The drug resistance of Huh7/s cells was maintained by the addition of 4.3 μM sorafenib. Sorafenib (HY-10201) and 1,25(OH)_2_D_3_ (calcitriol, HY-10002) were procured from MedChemExpress (Monmouth Junction, NJ, USA).

### 2.9. CCK8 Assay and Combination Index Calculations

Cell proliferation assays were performed using the CCK-8 assay [27]. Huh7/s cells were seeded into 96-well plates at a density of 1 × 10^5^ cells per well. After cell attachment, Huh7/s cells were treated with sorafenib or 1,25(OH)_2_D_3_ individually or in combination for 48 h. Following the addition of 10 μL of CCK-8 reagent (HY-K0301, MedChemExpress) to each well, plates were further incubated for 2 h. Optical density (OD) values of each well were measured at 450 nm using a spectrophotometer. The half-maximal inhibitory concentration (IC_50_) of sorafenib or 1,25(OH)_2_D_3_ was calculated using GraphPad 9.5 Software (San Diego, CA, USA), and cell proliferation curves were generated. The synergistic index of the drugs (represented by the combination index q-value) was calculated using Kim’s formula [28,29,30,31], where EA and EB denote the inhibition rates of proliferation when drugs A and B are used individually, and E(A + B) denotes the inhibition rate when drugs A and B are used in combination. A q-value > 1.15 indicates synergism, q = 0.85~1.15 indicates an additive effect, and q < 0.85 indicates antagonism.

$$q=\frac{E(A+B)}{EA+(1-EA)\times EB}$$

### 2.10. Cell Clone Formation Experiments

Huh7/s cells were seeded into 6-well plates at a density of 5 × 10^2^ cells per well. After 4 days of culture, the cells were treated with 4.3 μM sorafenib, 5 μM 1,25(OH)_2_D_3_, or their combination for 48 h. Subsequently, the medium was replaced with complete fresh medium and incubated for 12 days. Cells were washed twice with phosphate-buffered saline (PBS), fixed with 4% paraformaldehyde for 30 min, and stained with crystal violet solution after two additional PBS washes. Plates were air-dried and photographed for colony visualization, and the number of visible colonies was quantified using ImageJ 2 software (National Institutes of Health, Bethesda, MD, USA).

### 2.11. Apoptosis and Cell Cycle Analysis

Huh7/s cells were treated with 4.3 μM sorafenib and 5 μM 1,25(OH)_2_D_3_, either alone or in combination, for 48 h. Following treatment, the cells were harvested, washed with PBS, and subjected to Annexin V-FITC/PI staining according to the manufacturer’s instructions. Labeled cells were analyzed by flow cytometry to assess apoptotic changes in Huh7/s cells. This experiment was independently repeated three times.

Additionally, Huh7/s cells were treated with 4.3 μM sorafenib and 5 μM 1,25(OH)_2_D_3_ alone or in combination for 48 h. Cells were collected and washed with PBS, then resuspended in pre-cooled 70% ethanol and fixed at −20 °C overnight. After centrifugation to remove the ethanol residue and a subsequent wash with PBS, the cells were stained with propidium iodide (PI) and incubated at 4 °C for 30 min in the dark. Cell cycle phase distribution was assessed by flow cytometry, with three independent experiments conducted.

### 2.12. Monodansylcadaverine (MDC) Staining

Huh7/s cells were seeded into 12-well plates and treated with 4.3 μM sorafenib, 5 μM 1,25(OH)_2_D_3_, or their combination for 48 h. Following treatment, the medium was aspirated, and cells were washed once with 1× wash buffer. An appropriate volume of monodansyl cadaverine (MDC) staining solution was then added, and cells were incubated at room temperature for 15–45 min in the dark. After staining, cells were washed twice with 1× wash buffer and immediately observed under a fluorescence microscope (excitation filter: 355 nm; emission filter: 512 nm) for image acquisition.

### 2.13. Transmission Electron Microscopy

After the treatment of Huh7/s cells with 4.3 μM sorafenib and 5 μM 1,25(OH)_2_D_3_ alone or in combination, respectively, for 48 h, the cells were fixed in 2.5% glutaraldehyde solution and refixed in 1% osmium tetroxide. They were then dehydrated in 30%, 50%, 70%, 80%, 90%, 95% and 100% acetone. Embedding was carried out using Epon-812 pure embedding agent. Ultrathin sections of 60~90 nm in size were made in an ultrathin sectioning machine and fished onto a copper mesh and stained, firstly with uranyl acetate for 10~15 min, and then with lead citrate for 1~2 min at room temperature. Finally, the sections were observed under an electron microscope (HITACHI).

### 2.14. Western Blot

After 48 h of treatment with sorafenib and 1,25(OH)_2_D_3_, either alone or in combination, the cells were lysed with a radioimmunoprecipitation assay (RIPA) lysis buffer, and the supernatant was collected following centrifugation. The BCA protein assay kit, obtained from Biyuntian Biotechnology Research Institute (Shanghai, China), was used to estimate the protein concentration of the cells. Subsequently, SDS-PAGE electrophoresis was performed, and the proteins were transferred to a PVDF membrane, which was then blocked on a shaker at room temperature with 5% skimmed milk powder for 2 h. After blocking, the membrane was washed and incubated overnight at 4 °C with antibodies against key target proteins, diluted according to the manufacturer’s instructions. The primary antibodies used included anti-FOXO3A (ab109629, Abcam, Cambridge, UK), anti-p-FOXO3A (ab154786, Abcam, Cambridge, UK), anti-LC3B (2775S, CST, Danvers, MA, USA), anti-FOXM1 (13147-1-AP, Proteintech, Wuhan, China), anti-Bcl-2 (60178-1-IG, Proteintech, Wuhan, China), anti-Bax (60267-1-IG, Proteintech, Wuhan, China), anti-P62 (18420-1-AP, Proteintech, Wuhan, China), and anti-Beclin-1 (11306-1-AP, Proteintech, Wuhan, China). On the following day, the membrane was washed again, and HRP-labeled goat anti-rabbit IgG secondary antibody was added, followed by incubation at room temperature for 1 h. ECL detection was performed, and the results were analyzed using ImageJ software.

### 2.15. Statistical Methods

For the statistical examinations, the GraphPad software was employed. The data are showcased in the format of mean ± standard deviation (X ± SD). When it came to comparing the differences between two groups, the *t*-test was utilized. In contrast, for evaluating the differences among multiple groups, a one-way ANOVA was carried out. Statistical significance was determined at a significance level of *p* < 0.05.

## 3. Results

### 3.1. 1,25(OH)_2_D_3_ Target Prediction and Screening of Sorafenib Resistance Genes in Hepatocellular Carcinoma

**Figure 1:**
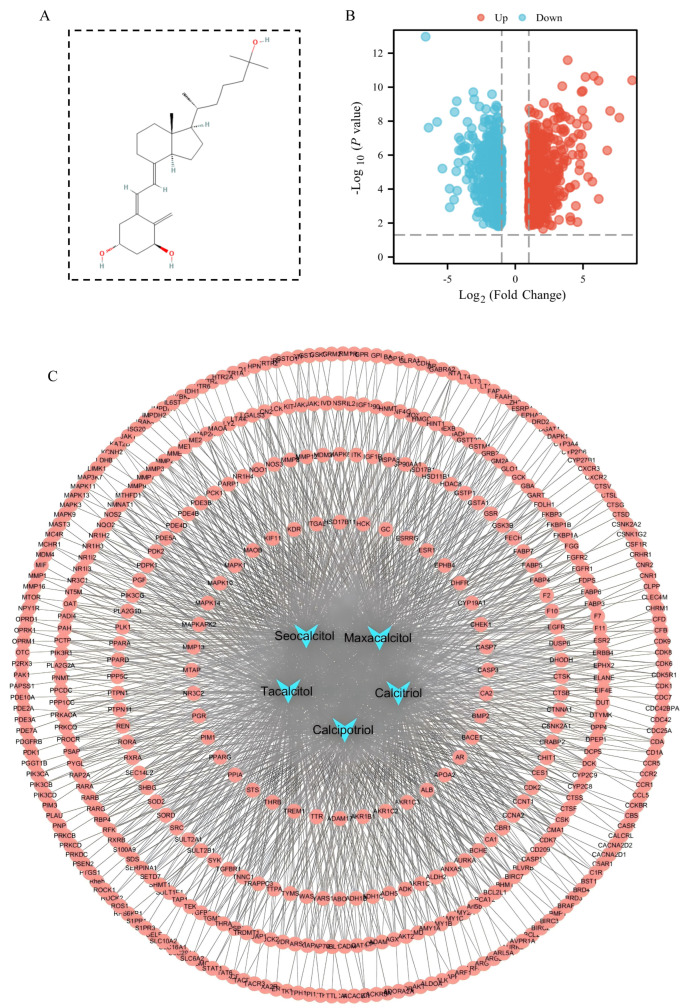
1,25(OH)_2_D_3_ target prediction and screening of sorafenib resistance genes in hepatocellular carcinoma. (**A**) Chemical structure of 1,25(OH)_2_D_3_; (**B**) volcano diagram of sorafenib resistance differential genes in hepatocellular carcinoma; (**C**) compound–target network diagram. Blue nodes represent compounds, red nodes represent compound-predicted targets, and connecting lines represent interactions between compounds and predicted targets.

The chemical structure of 1,25(OH)_2_D_3_ is depicted in Figure 1A, and its derivative structures are detailed in Table S1. Three target prediction databases—Pharm Mapper, Swiss Target Prediction, and SEA—were employed to analyze 1,25(OH)_2_D_3_ and its derivatives. After eliminating duplicates, 730 predicted targets were identified (Table S2). Network analysis and visualization of these targets were conducted using Cytoscape v3.7.2, resulting in the construction of a compound–target network diagram (Figure 1C). The dataset GSE94550 was retrieved from the GEO database and analyzed using GEO2R to identify differentially expressed genes. A total of 1167 liver cancer sorafenib resistance genes were identified based on criteria (*p* < 0.05, |logFC| > 1), comprising 551 up-regulated genes and 616 down-regulated genes (Table S3). Visualization of these differential genes was performed, and a gene volcano plot is illustrated in Figure 1B.

### 3.2. Identification of Potential Targets for Sorafenib Resistance in 1,25(OH)_2_D_3_-Treated HCC

**Figure 2:**
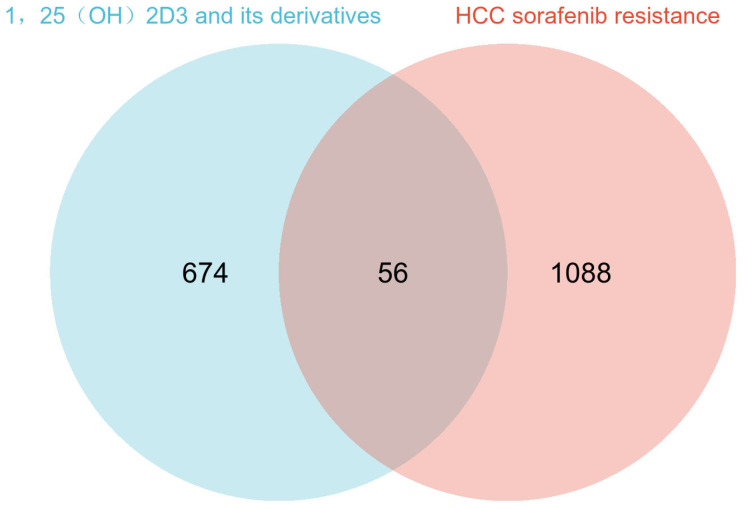
Venn analysis of 1,25(OH)_2_D_3_ and its derivatives’ predictive targets and sorafenib resistance genes in hepatocellular carcinoma.

To identify potential targets through which 1,25(OH)_2_D_3_ may act on sorafenib resistance in hepatocellular carcinoma (HCC), we analyzed the overlap between predicted targets of 1,25(OH)_2_D_3_ and its derivatives, and sorafenib resistance targets in HCC using Venn diagrams. Figure 2 illustrates that a total of 56 targets were found to overlap between the predicted targets of 1,25(OH)_2_D_3_ and its derivatives, and sorafenib-resistant targets in HCC.

### 3.3. GO and KEGG Analysis

**Figure 3:**
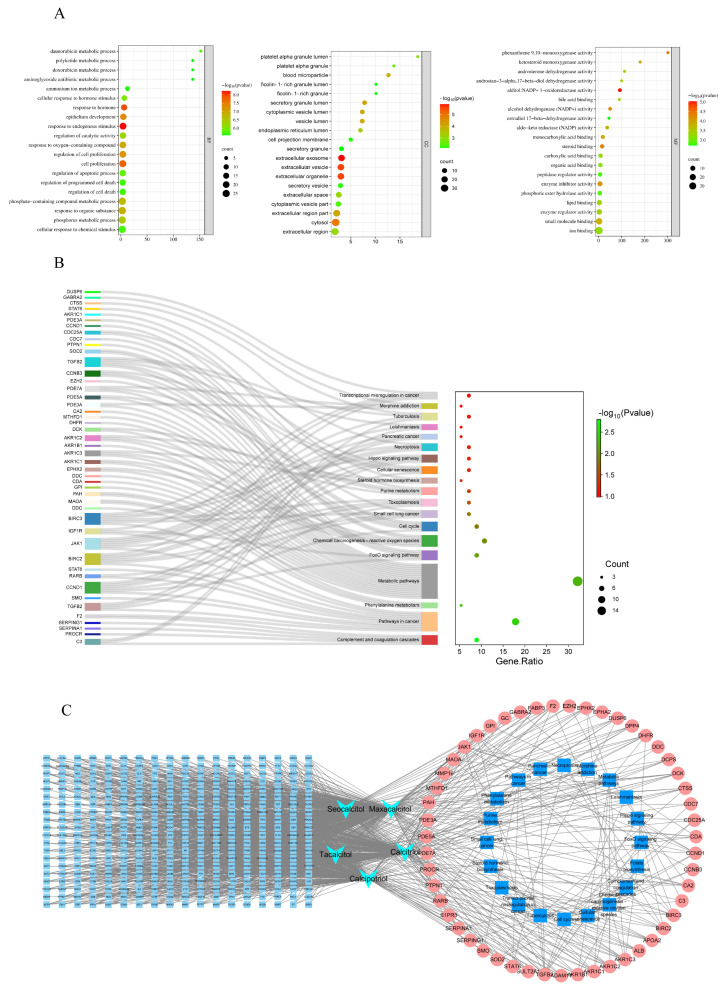
GO and KEGG enrichment analysis of 56 key targets. (**A**). Biological process (bp), cellular component (cc) and molecular function (MF) enrichment analysis diagram; (**B**). KEGG analysis of 56 key targets. (**C**). Construction of compound–target–pathway network diagram.

In order to thoroughly explore the molecular mechanism of 1,25(OH)_2_D_3_ key targets acting on sorafenib resistance in hepatocellular carcinoma, GO analysis and KEGG analysis were performed in this study. The top 20 GO terms of BP, CC and MF (*p* < 0.05) are shown in Figure 3A; BP was mainly involved in phosphorus metabolism, protein modification and the regulation of molecular function, while CC was mainly related to the membrane region, plasma membrane region, cytoplasm, etc. MF was mainly involved in macromolecular complexes, metal ion binding and other biological processes. Meanwhile, KEGG pathway enrichment analysis showed (Figure 3B) that these targets were mainly related to signaling pathways such as pathways in cancer, the FoxO signaling pathway (*p* < 0.05, FDR < 0.25), etc. In addition, a compound–target–pathway network was constructed in this study (Figure 3C), and the results showed that these targets play a role in the drug resistance process of hepatocellular carcinoma through multiple pathways. By integrating literature studies and enrichment results, we focused on the FoxO signaling pathway. Activation of the FoxO signaling pathway plays a crucial role in tumorigenesis, chemotherapy resistance, and metabolic regulation. Additionally, FoxO is a significant class of transcription factors that participates in various essential biological processes, including the cell cycle, cell proliferation, apoptosis, and the response to oxidative stress [13].

### 3.4. Molecular Docking of Small Molecule Models with FOXO3A

**Figure 4:**
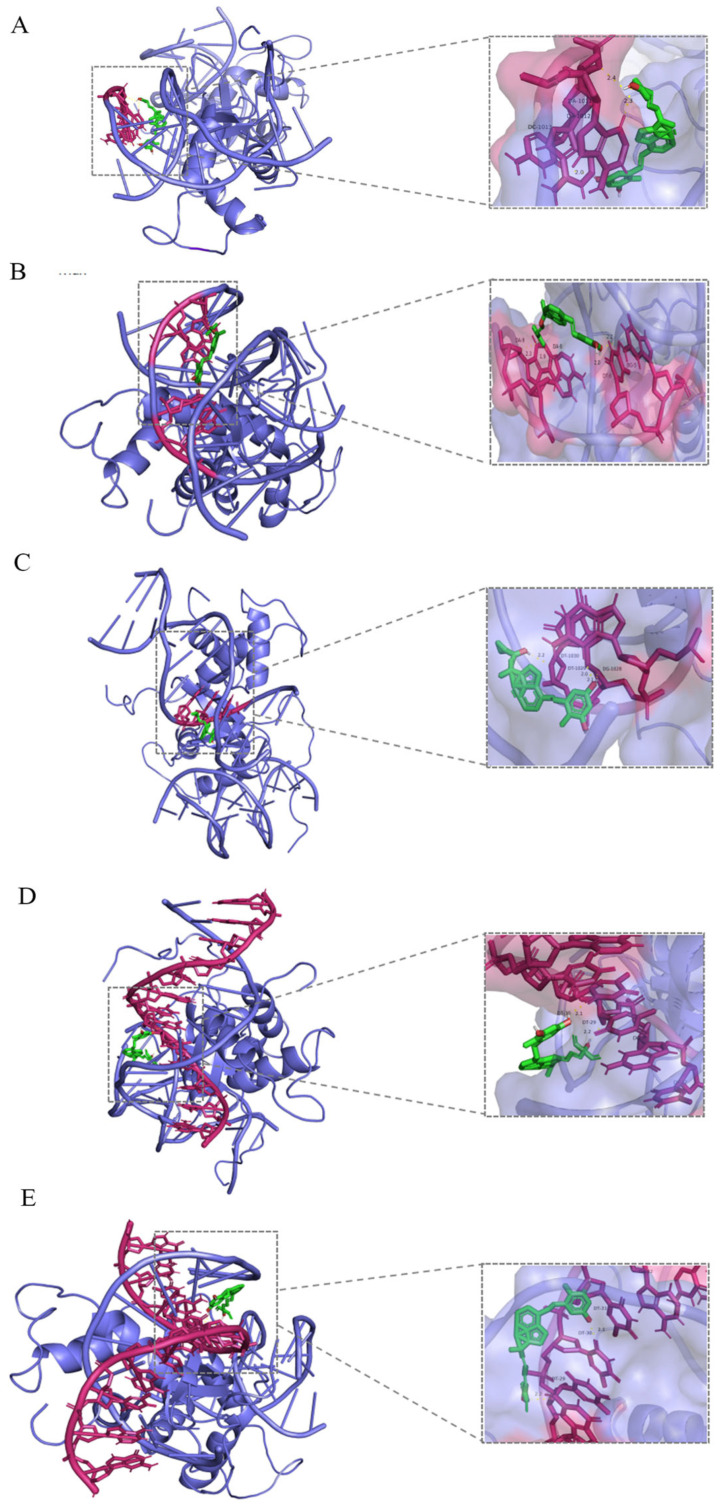
Molecular docking models of 1,25(OH)_2_D_3_ and its derivatives with FOXO3A. (**A**) Molecular docking model of 1,25(OH)_2_D_3_ with FOXO3A; (**B**) molecular docking model of Maxacalcitol with FOXO3A; (**C**) molecular docking model of Calcipotriol with FOXO3A; (**D**) molecular docking model of Seocalcitol with FOXO3A; (**E**) molecular docking model of Tacalcitol with FOXO3A (Green represents small molecule compounds, while red and blue represent proteins).

A molecular docking model of 1,25(OH)_2_D_3_ and its derivatives (Maxacalcitol, Calcipotriol, Seocalcitol, Tacalcitol) with FOXO3A was constructed using AutoDock software. The results indicated that 1,25(OH)_2_D_3_ and its derivatives interacted with amino acid residues at the binding site of FOXO3A through hydrogen bonding interactions (Figure 4). Furthermore, the binding energies of FOXO3A with 1,25(OH)_2_D_3_ and its derivatives were all below −7 kcal/mol (Table 1), suggesting a high probability of stable binding between FOXO3A and these small molecule models.

### 3.5. Molecular Dynamics Simulations

**Figure 5:**
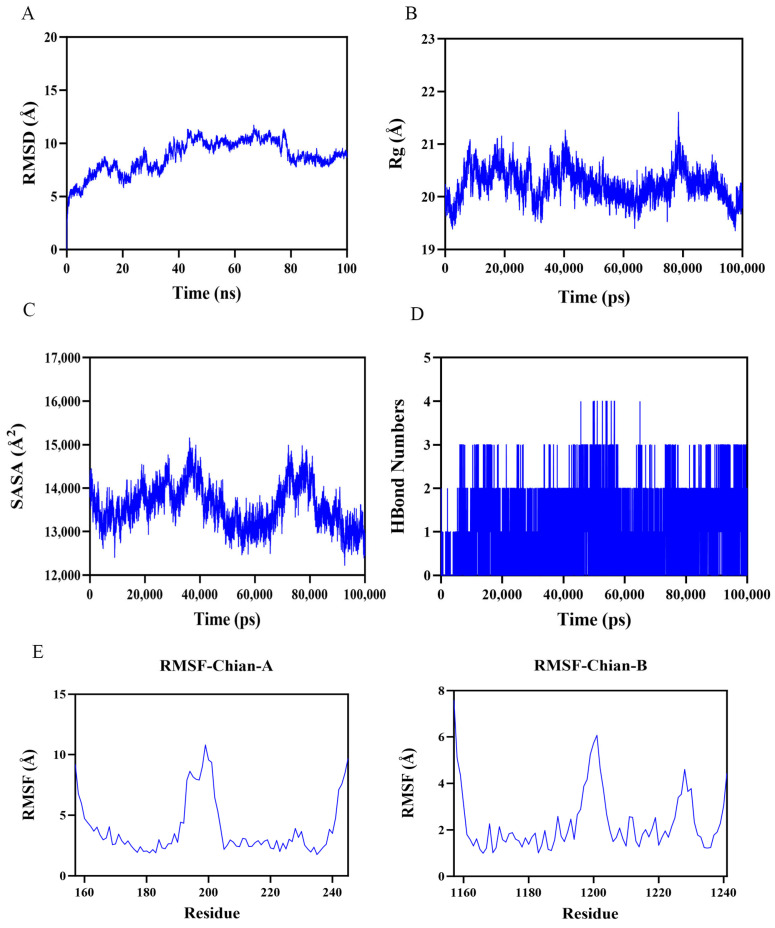
Molecular dynamics simulation results showing the stability and conformational changes of the ligand–protein complexes in 100 ns simulations. (**A**). RMSD curves for the stability of the 1,25(OH)_2_D_3_–FOXO3A complex; (**B**) Rg changes in the 1,25(OH)_2_D_3_–OXO3A complex; (**C**) 1,25(OH)_2_D_3_–FOXO3A complex SASA variation; (**D**) hydrogen bonding number in 1,25(OH)_2_D_3_–FOXO3A complex; (**E**) PMSF curves of 1,25(OH)_2_D_3_–FOXO3A complexes.

To further verify the reliability of molecular docking, we performed 100 ns molecular dynamics simulations for the docking results with the highest binding energies. The root mean square deviation (RMSD) is a good indicator of the conformational stability of proteins and ligands, as well as a measure of the degree of deviation of the atomic positions from their starting positions. The smaller the deviation, the better the conformational stability. Therefore, the ligand–protein complexes were evaluated using RMSD over a 100 ns simulation period. As shown in Figure 5A, the complex systems all reached equilibrium after 80 ns and eventually fluctuated up and down at 2.7 Å. Thus, 1,25(OH)_2_D_3_ exhibited high stability when bound to the target protein FOXO3A. Further analysis revealed that the radius of gyration (Rg) value and solvent accessible surface area (SASA) of the complex system showed a slight fluctuation during the movement. It indicated that the target protein FOXO3A underwent a conformational change due to the binding of 1,25(OH)_2_D_3_ (Figure 5B,C). In addition, stable hydrogen bonds were formed between 1,25(OH)_2_D_3_ and the target protein during the kinetic process, and the number fluctuated between 0 and 4 (Figure 5D), which indicated that 1,25(OH)_2_D_3_ had a good hydrogen bonding interaction with the target protein. Root mean square fluctuation (RMSF) can indicate the flexible size of amino acid residues in proteins. As shown in Figure 5E, the RMSF values of complex systems are relatively low (mostly below 6 Å), so they are less flexible and more stable. In summary, the complex systems are stable in binding and the complexes have good hydrogen bonding. Therefore, 1,25(OH)_2_D_3_ binds well to the target protein FOXO3A.

### 3.6. 1,25(OH)_2_D_3_ in Combination with Sorafenib Inhibits the Proliferation of Huh7/s Cells

**Figure 6:**
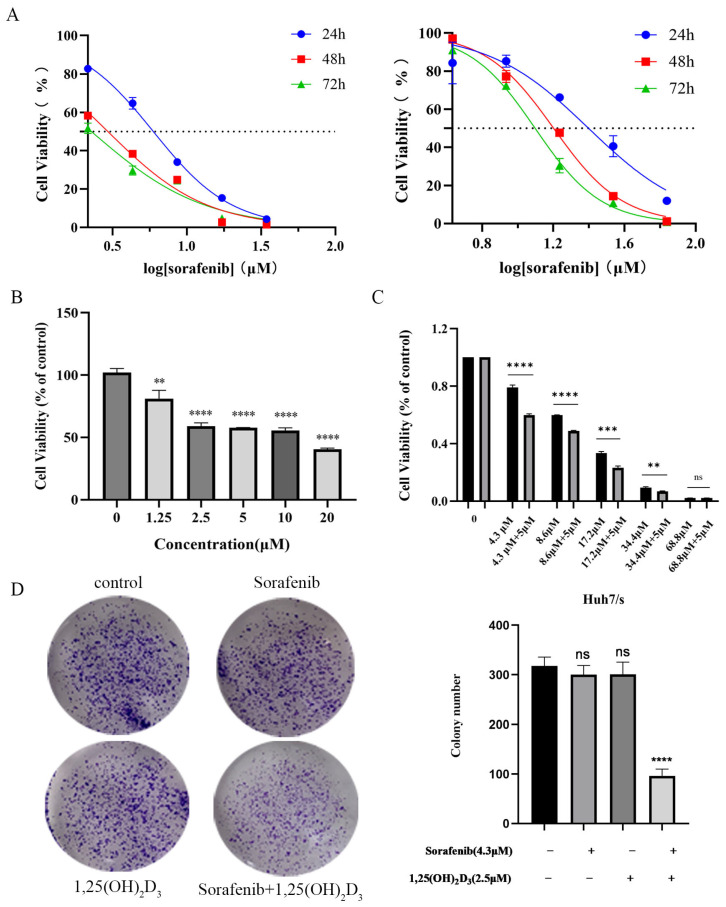
1,25(OH)_2_D_3_ combined with sorafenib inhibited the proliferation of Huh7/s cells. (**A**) Effect of sorafenib on proliferation of Huh7 and sorafenib-resistant Huh7/s (Huh7/s) cells; (**B**) 1,25(OH)_2_D_3_ inhibition of the proliferation of Huh7/s cells; (**C**) inhibition of the proliferation of Huh7/s cells by 1,25(OH)_2_D_3_ combined with sorafenib; (**D**) inhibition of the clone-forming ability of Huh7/s cells by 1,25(OH)_2_D_3_ combined with sorafenib. ** *p* < 0.01, *** *p* < 0.001, and **** *p* < 0.0001, ns, not significant.

The cell viability data for parental Huh7 and sorafenib-resistant Huh7/s cells are presented in Figure 6A. The resistance indices of Huh7/s cells at 24, 48, and 72 h were 4.22, 4.6, and 5.68 times higher than those of Huh7, respectively, demonstrating the sorafenib resistance of Huh7/s cells. Subsequently, Huh7/s cells were treated with varying concentrations of 1,25(OH)_2_D_3_ (1.25, 2.5, 5, 10, 20 μM) for 48 h. The results shown in Figure 6B indicate that 1,25(OH)_2_D_3_ significantly inhibited the proliferation of Huh7/s cells, with all differences being statistically significant (*p* < 0.05). The IC_50_ value of 1,25(OH)_2_D_3_ was determined to be 10.38 μM (Figure S1). Further investigations involved combining 5 μM 1,25(OH)_2_D_3_ with different concentrations of sorafenib (4.3, 8.6, 17.2, 34.4, 68.8 μM). As depicted in Figure 6C, the cell viability in the combination group was markedly reduced compared to that in the sorafenib-only group. Additionally, q-values were calculated for the combination of 5 μM 1,25(OH)_2_D_3_ with different sorafenib concentrations (Table 2). The q-value analysis identified that the combination of 5 μM 1,25(OH)_2_D_3_ with 4.3 μM Sorafenib exhibited the highest efficacy; thus, this concentration combination was selected for subsequent experiments. To further evaluate the inhibitory effects of this combination on Huh7/s cells, a colony formation assay was conducted. The results depicted in Figure 6D demonstrate a significant reduction in the number of cell colonies in the combination treatment group compared to the sorafenib-only group. These findings strongly suggest that 1,25(OH)_2_D_3_ holds promise as a potential candidate for enhancing the chemosensitivity of sorafenib in hepatocellular carcinoma.

### 3.7. 1,25(OH)_2_D_3_ in Combination with Sorafenib Promotes Apoptosis and Cycle Blockade in Huh7/s Cells

**Figure 7:**
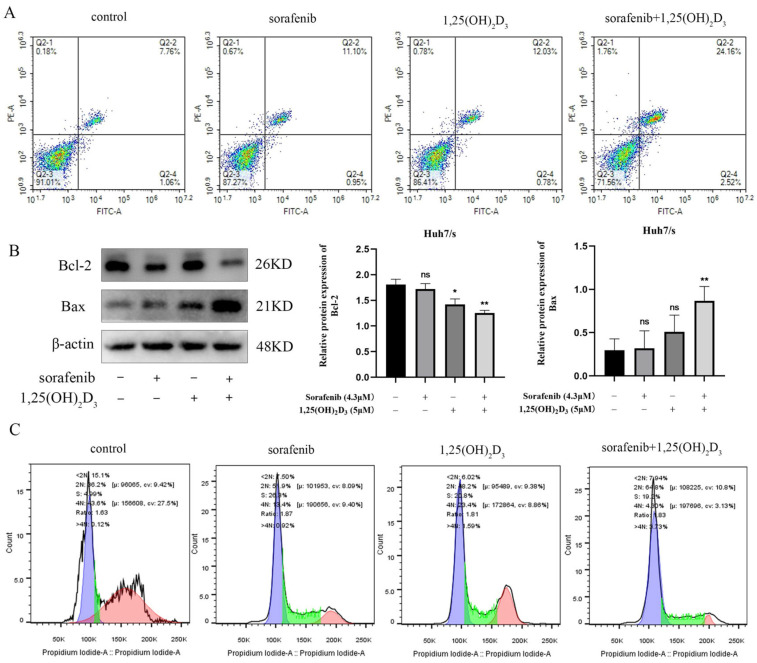
Effects of 1,25(OH)_2_D_3_ combined with sorafenib on the proliferation and cycle of Huh7/s cells. (**A**) Flow cytometry to detect the effects of 1,25(OH)_2_D_3_ on the apoptosis of Huh7/s cells; (**B**) changes in apoptotic protein levels of Huh7/s cells treated with 1,25(OH)_2_D_3_ combined with sorafenib for 48 h detected by Western blot; (**C**) flow cytometry for detecting the effect of 1,25(OH)_2_D_3_ combined with sorafenib on the Huh7/s cell cycle. * *p* < 0.05, ** *p* < 0.01, ns, not significant.

Flow cytometry was employed to observe the impact of 1,25(OH)_2_D_3_ combined with sorafenib on apoptosis in Huh7/s cells. The results indicated a significant increase in the apoptotic subpopulation of Huh7/s cells when treated with the combination compared to sorafenib alone (Figure 7A). Subsequently, we assessed the levels of apoptosis-related proteins via Western blot analysis. Figure 7B demonstrates a significant upregulation of the pro-apoptotic protein Bax and a notable downregulation of the apoptosis-inhibiting protein Bcl-2 in response to the combination treatment. Moreover, sorafenib is known to specifically block the G1/G0 phase of the cell cycle. Therefore, we also evaluated the effect of 1,25(OH)_2_D_3_ in combination with sorafenib on the cell cycle distribution of Huh7/s cells. Flow cytometry analysis revealed an increased G1/G0 subpopulation upon treatment with the combination (Figure 7C). Taken together, these findings suggest that 1,25(OH)_2_D_3_ may enhance chemosensitivity by promoting apoptosis and cell cycle arrest in Huh7/s cells.

### 3.8. 1,25(OH)_2_D_3_ Combined with Sorafenib Inhibits Autophagy in Huh7/s Cells

**Figure 8:**
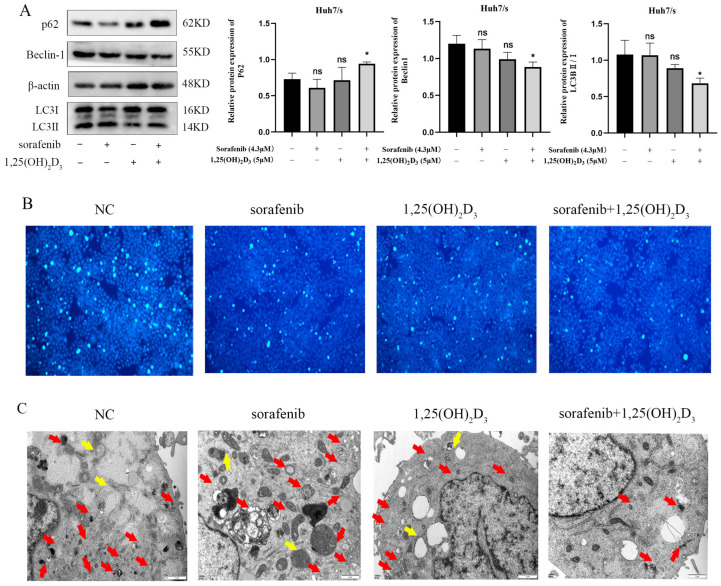
Effect of 1,25(OH)_2_D_3_ in combination with sorafenib on autophagy in Huh7/s cells. (**A**) Changes in autophagy protein levels detected by Western blot after treatment of Huh7/s cells for 48 h with 1,25(OH)_2_D_3_ in combination with sorafenib; quantitative bar graphs were obtained by normalization using Image J. (**B**) 1,25(OH)_2_D_3_ in combination with sorafenib after treatment of Huh7/s cells for 48 h; autophagic vesicles were stained by the monodansyl cadaverine (MDC) method (×100, scale is 100 μm). (**C**) Treatment of Huh7/s cells for 48 h, with 1,25(OH)_2_D_3_ combined with sorafenib, by transmission electron microscopy (scale bar: 1 μm). (Red arrows indicate autophagosomes; yellow arrows indicate autolysosomes). * *p* < 0.05, ns, not significant.

Autophagy plays a crucial role in chemoresistance, and its inhibition represents a strategy to mitigate sorafenib resistance. To investigate this, we assessed autophagy-related proteins using Western blot analysis. As depicted in Figure 8A, the expression of the autophagy marker P62 increased, while the levels of Beclin-1 and LC3B decreased compared to the control group, suggesting that the combination treatment is more effective in suppressing autophagy than single-agent therapy. Staining of autophagic vesicles using monodansyl cadaverine (MDC) (Figure 8B) and observation of autophagy using transmission electron microscopy (Figure 8C) further confirmed that the association could inhibit autophagy.

### 3.9. Effect of 1,25(OH)_2_D_3_ Combined with Sorafenib on FOXO3A/FOXM1 Axis

**Figure 9:**
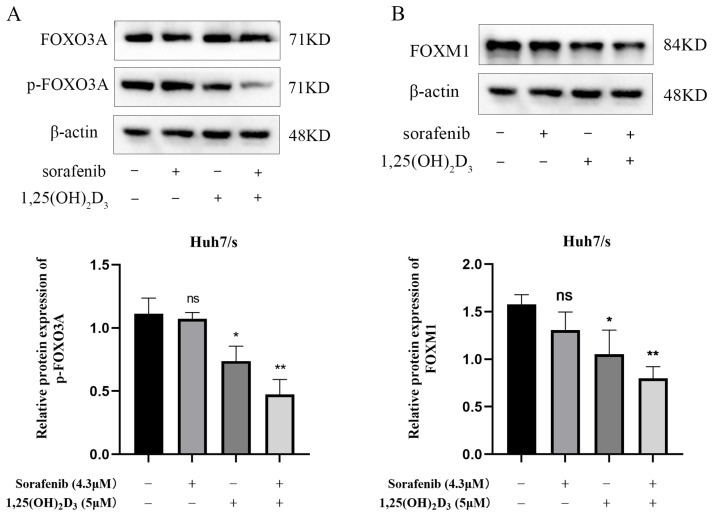
Effect of 1,25(OH)_2_D_3_ combined with sorafenib on FOXO3A/FOXM1 axis. (**A**,**B**). Changes in p-FOXO3A and FOXM1 detected by Western blot after treatment of Huh7/s cells for 48 h with 1,25(OH)_2_D_3_ combined with sorafenib. * *p* < 0.05, ** *p* < 0.01, ns, not significant.

**Figure 10:**
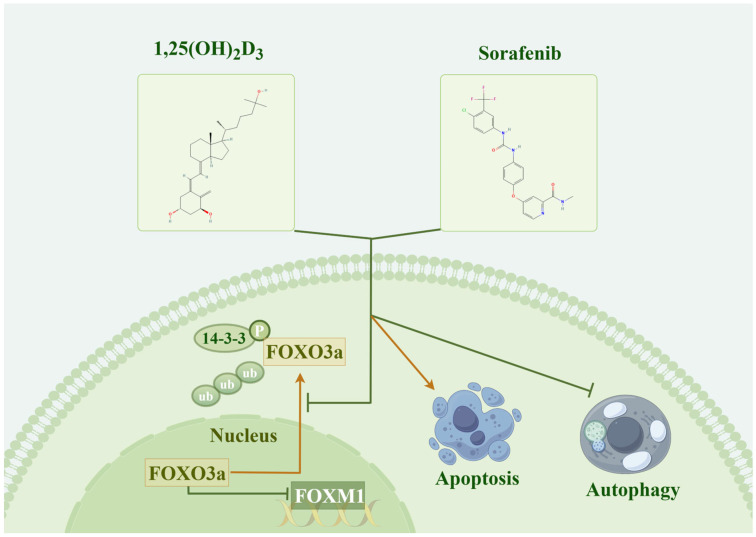
Molecular mechanism diagram.

FOXO3A has been implicated in tumor development and chemoresistance, which is closely linked to apoptosis and autophagy. Network pharmacology and molecular docking results highlighted significant enrichment of the FoxO signaling pathway. Moreover, FOXO3A exhibited strong binding affinity with both 1,25(OH)_2_D_3_ and sorafenib. We found that the level of FOXO3A phosphorylation was significantly reduced in the co-treated group compared with the control group by a Western blot assay (Figure 9A). FOXM1, a downstream target of FOXO3A closely associated with chemotherapy resistance, also showed reduced expression upon drug combination, as indicated by Western blotting (Figure 9B). These findings suggest that 1,25(OH)_2_D_3_ enhances sorafenib chemosensitivity in hepatocellular carcinoma by deactivating the FOXO3A/FOXM1 axis. The general mechanism diagram of this study is shown in the Figure 10.

## 4. Discussion

The development of chemotherapy resistance has long been a significant challenge in cancer treatment. Approximately 90% of cancer-related deaths are attributed to primary or acquired drug resistance and subsequent metastasis [32]. Despite this, the molecular mechanisms underlying tumor drug resistance remain largely unclear. How to effectively improve the sensitivity of tumors to chemotherapeutic drugs or avoid drug resistance has become an urgent problem worldwide.

In recent years, combination therapy to overcome resistance to single chemotherapeutic agents has garnered increasing attention. Numerous studies have demonstrated that osteotriol (1,25(OH)_2_D_3_), a biologically active metabolite of vitamin D synthesized through two-step metabolism in the liver and kidney, plays pivotal roles in multiple signaling pathways governing proliferation, apoptosis, differentiation, inflammation, invasion, angiogenesis, and metastasis. Consequently, it possesses the potential to influence tumorigenesis and growth [33]. Additionally, studies have demonstrated that 1,25(OH)_2_D_3_ interacts with antimetabolites (e.g., 5-fluorouracil, gemcitabine) and various other drugs, significantly enhancing the therapeutic efficacy of individual chemotherapeutic agents. These include antimetabolites (e.g., 5-fluorouracil, gemcitabine), platinum compounds (e.g., cisplatin, oxaliplatin, carboplatin), paclitaxel analogs (e.g., paclitaxel, docetaxel), and tyrosine kinase inhibitors (e.g., gefitinib, erlotinib) [8]. Chronic inflammation has been well documented as a critical driver in the pathogenesis and progression of numerous malignancies [34]. Emerging evidence demonstrates that 1,25(OH)_2_D_3_ exerts potent anti-inflammatory properties, positioning it as a promising chemopreventive agent for diverse cancer types [6,35,36,37]. Maintenance of optimal vitamin D status through supplementation in deficient individuals may confer chemopreventive benefits and reduce oncogenic risks. Therapeutic administration of 1,25(OH)_2_D_3_, either as monotherapy or in combination with active anticancer agents, could potentially inhibit carcinogenesis and/or decelerate tumor progression. For instance, the results obtained by Ye Li et al. [38], utilizing data from breast cancer patients, human tamoxifen (TAM)-resistant breast cancer cell models, and animal models, collectively demonstrate that vitamin D (VitD) can suppress TAM-induced pro-survival autophagy and restore the sensitivity of TAM-resistant breast cancer cells to TAM therapy, while also inhibiting the development of murine breast cancer in in vivo models. Furthermore, higher vitamin D receptor (VDR) levels correlate with improved prognosis in TAM-treated patients, suggesting that VitD may prevent or reverse TAM resistance in breast cancer patients. Studies by Zhirong Jia et al. [39] revealed that the combination of 1,25(OH)_2_D_3_ and gefitinib significantly reduces cell viability, proliferation, and tumor progression in both PC-9/GR cells and PC-9/GR xenograft tumor models compared to monotherapy, offering a promising strategy to enhance gefitinib’s cytotoxicity. Current evidence also highlights vitamin D’s potential dual role in treating and preventing glioblastoma [40,41,42,43], supporting the clinical application of vitamin D or VDR as novel biomarkers in this context. Consequently, early-stage intervention with 1,25(OH)_2_D_3_ therapy may improve sorafenib’s cytotoxic effects, prevent drug resistance, and prolong chemotherapy duration. Notably, 1,25(OH)_2_D_3_ exhibits superior safety and tolerability profiles compared to regorafenib, a second-line therapeutic agent administered following sorafenib treatment progression. While regorafenib demonstrates modest survival benefits, its clinical utility is limited by significant toxicities, including hand–foot skin reactions, hypertension, gastrointestinal disturbances, and hepatotoxicity [44], which may compromise patient quality of life and treatment adherence. Clinical studies have shown that super-physiological doses of 1,25(OH)_2_D_3_ mainly induce dose-dependent hypercalcemia, but this adverse reaction can be effectively controlled through strict serum monitoring and intermittent administration [45]. Furthermore, novel 1,25(OH)_2_D_3_ analogs have been engineered to minimize calcitropic side effects while preserving or enhancing the compound’s therapeutic efficacy. Some of the existing analogues have tissue-specific effects and low calcification side effects, and can be administered at higher doses compared with the parent compounds [46,47]. Seocalcitol had potent antiproliferative effects in vitro and significantly decreased tumor growth in vivo in animal models of head and neck squamous cell carcinoma [48]. Inecalcitol is a sidechain analog of calcitriol and has shown more potently decreased tumor growth in various cancer models, including breast [49], squamous cell [50], and prostate [51] cancer models, compared with calcitriol. Furthermore, in mouse experiments, inecalcitol induced tumor regression without significantly affecting serum calcium levels. Reasonable utilization of these structural derivatives may thus optimize pharmacokinetic stability and safety profiles without compromising the antitumor benefits inherent to 1,25(OH)_2_D_3_ signaling pathways. In our study, 1,25(OH)_2_D_3_ combined with sorafenib exhibited antiproliferative and pro-apoptotic effects on hepatocellular carcinoma sorafenib-resistant cells (Huh7/s), suggesting that 1,25(OH)_2_D_3_ has the potential to enhance the sensitization of Huh7/s to sorafenib, and may serve as a potential agent to improve the response to chemotherapy. Therefore, the potential mechanism of 1,25(OH)_2_D_3_ in the sensitization of HCC chemotherapy needs to be further investigated.

In our work, the action target of 1,25(OH)_2_D_3_ was analyzed using Wayne’s analysis against genes associated with hepatocellular carcinoma sorafenib resistance, identifying intersecting genes as potential targets of 1,25(OH)_2_D_3_ for sensitizing hepatocellular carcinoma to sorafenib chemotherapy. Subsequently, GO and KEGG enrichment analyses were conducted on these potential sensitization targets to identify pathways through which 1,25(OH)_2_D_3_ may enhance chemotherapy. Finally, a chemo–target–pathway network was constructed. Furthermore, the underlying molecular mechanisms of 1,25(OH)_2_D_3_ in enhancing chemosensitivity were preliminarily validated through in vitro experiments. KEGG pathway analysis revealed that 1,25(OH)_2_D_3_-mediated chemosensitization involves the Hippo signaling pathway, FoxO signaling pathway, and JAK-STAT signaling pathway, all crucial in tumor development [52]. FOXO3A belongs to the FOXO subfamily of forkhead transcription factors, which plays a crucial role in cancer progression, drug resistance, etc. FOXO3A is considered a tumor suppressor, but it is frequently inactivated in cancer cell lines by the nuclear translocation of the FOXO3A protein. FOXO3A is phosphorylated by Akt, ERK, SGK, IKKβ, and IKBKE, among others. Dysregulation of these kinases is frequently observed in different types of cancer, promoting nucleoplasmic translocation and/or the ubiquitin/proteasome-dependent degradation of FOXO3A, thereby promoting cancer progression [53]. In this study, we evaluated the interaction of 1,25(OH)_2_D_3_ and its derivatives with FOXO3A using a molecular docking approach. Molecular dynamics simulation analysis was used to further show that 1,25(OH)_2_D_3_ binds significantly to the target protein FOXO3A. The results indicated that both compounds exhibited strong docking activity with FOXO3A, suggesting that they can bind stably to the protein. Since the transcriptional activity of FOXO3A is regulated by its nuclear translocation, which can be inhibited by kinase-mediated phosphorylation, we examined the level of phosphorylation FOXO3A. Compared to treatment with either drug alone, combined treatment of 1,25(OH)_2_D_3_ and sorafenib significantly inhibited the level of phosphorylated FOXO3A protein. Studies have demonstrated that the FOXO3A/FOXM1 axis plays a critical role in tumor progression and chemoresistance. FOXM1 is the direct transcription target of FOXO3A [54,55,56]. Reactivation of FOXO3A or suppression of FOXM1 in cancer cells reduces DNA repair capacity and cell survival rates while augmenting cell death and enhancing the efficacy of DNA-damaging anticancer therapies [57,58]. For instance, Junnan Li et al. [59] established a novel and promising strategy to overcome acquired resistance to erlotinib by inducing cell cycle arrest at the G1/S phase via modulation of the FOXO3A/FOXM1 axis. Findings from Hao Liu et al. [60] further revealed the pivotal role of the DNMT1/FOXO3a/FOXM1/SOX2 signaling cascade in regulating breast cancer stem cell (BCSC) properties, providing a rationale for developing therapeutics targeting this pathway to suppress BCSC-driven drug resistance. Additional studies report that the combination of trametinib and the glutamine transporter inhibitor V-9302 increases pyroptosis and cell cycle arrest by modulating the FOXO3A/FOXM1 axis and autophagy [54]. Therefore, we also examined FOXM1 expression and found that the drug combination reduced FOXM1 levels. These findings suggest that 1,25(OH)_2_D_3_ may influence sorafenib resistance in hepatocellular carcinoma by modulating the FOXO3A/FOXM1 axis, indicating that targeting FOXO3A and FOXM1 could be a promising molecular therapeutic strategy.

Additionally, dysregulation of FOXO3A has been shown to promote the development of drug resistance through multiple mechanisms, including the regulation of oncogenic signaling pathways, evasion of apoptosis, increased drug efflux, and induction of autophagy [61]. Cellular autophagy and apoptosis are crucial processes contributing to sorafenib resistance in HCC [62]. During apoptosis, members of the B-cell lymphoma-2 (Bcl-2) family play significant roles in regulating the mitochondrial apoptotic pathway and are classified into two main groups based on their effects: anti-apoptotic and pro-apoptotic proteins [63]. Current studies indicate that Bcl-2 proteins are linked to acquired sorafenib resistance and HCC proliferation [64,65]. Therefore, targeting the overexpression of Bcl-2 family proteins in HCC may provide an effective therapeutic strategy [66]. Multiple studies have demonstrated that FOXO3A regulates apoptosis in cancer cells by modulating the expression of apoptosis-related proteins [67,68,69,70]. For instance, Zhebin Dong et al. [71] revealed that miRNA-124-3p.1 regulates FOXO3A phosphorylation and deacetylation by targeting AKT2 and SIRT1, thereby sensitizing HCC cells to sorafenib-induced apoptosis. Additionally, Zhang et al. [67] reported that butein can activate FOXO3A, leading to the downregulation of Bcl-2 and upregulation of Bax, which enhances the sensitivity of cervical cancer cells to cisplatin (CDDP). Chemotherapy-induced autophagy has been identified as a pro-survival mechanism that contributes to drug resistance. Targeting autophagy is thus considered a promising therapeutic approach for cancer patients facing drug resistance. Studies have also highlighted that sorafenib-induced autophagy serves as a pro-survival response [72,73,74,75,76,77]. Furthermore, multiple studies have demonstrated that FOXO3A-mediated autophagy serves as a crucial mechanism underlying sorafenib resistance in HCC. For instance, the investigation by Chao Liang et al. [78] revealed that hypoxia-induced autophagy functions as a primary mechanism through which HCC cells develop sorafenib resistance, with FOXO3A playing a pivotal regulatory role in this hypoxia-triggered autophagic process both in vitro and in vivo. Research by Ziyou Lin et al. [79] has demonstrated that RNA strand m6A methylation modulates sorafenib resistance in HCC via FOXO3-mediated autophagy. Consequently, we examined apoptosis and autophagy in drug-resistant cells following combination drug treatment. As demonstrated by Western blot and flow cytometry experiments, the combination of 1,25(OH)_2_D_3_ and sorafenib induced apoptosis in Huh7/s cells by upregulating Bax and downregulating Bcl-2 expression. The combination’s ability to inhibit autophagy in Huh7/s cells was further confirmed by transmission electron microscopy and the detection of autophagy-related markers. These results suggest that the primary mechanism by which 1,25(OH)_2_D_3_ enhances the chemosensitivity of HCC to sorafenib may involve the FOXO3A/FOXM1 axis, regulating the proliferation and apoptosis of drug-resistant cells and the onset of cellular autophagy, thereby playing a role in reversing resistance to sorafenib in HCC.

Notably, a complex interplay exists between autophagy and apoptosis. Autophagy can promote or inhibit apoptosis, and apoptosis can also promote or inhibit autophagy [80,81,82,83]. Crucially, the dysregulation of this homeostatic crosstalk has been implicated in tumorigenesis [84]. In cytotoxicity studies, differential cellular responses are observed across cell types depending on pharmacological variables including dosage, concentration, and exposure duration. Our experimental findings demonstrate that co-administration of 1,25(OH)_2_D_3_ with sorafenib synergistically promotes apoptosis while suppressing autophagic flux in drug-resistant cells, effectively inhibiting proliferation and restoring chemosensitivity to sorafenib. This phenomenon may be attributed to autophagy impairment-triggered apoptotic activation in resistant cells, thereby curtailing neoplastic growth. However, as a tightly regulated catabolic process, autophagy exhibits dual oncogenic roles—acting as either a tumor suppressor or promoter depending on cellular context [85]. Therefore, systematic investigation into the apoptosis–autophagy interactions under combination therapy remains imperative for elucidating resistance mechanisms in malignant cells.

This study has several limitations that warrant consideration and future investigation. Primarily, it is imperative to systematically determine the optimal drug combination ratio to substantially enhance the clinical translational potential of this therapeutic regimen. Further validation using HCC drug-resistant cell models, coupled with rigorous preclinical validation in animal models and subsequent phase clinical trials, is essential to corroborate these findings and establish clinical applicability. Secondly, the mechanism by which 1,25(OH)_2_D_3_ enhances sorafenib chemosensitivity in hepatocellular carcinoma involves multiple targets and pathways. Although several key pathways have been identified in our experiments, these findings remain preliminary. Therefore, additional in-depth pharmacological investigations are warranted to fully elucidate these intricate mechanisms.

## 5. Conclusions

In summary, our study demonstrates that 1,25(OH)_2_D_3_ enhances the chemosensitivity of hepatocellular carcinoma to sorafenib and preliminarily elucidates the mechanisms underlying its chemosensitizing effects. This discovery provides critical insights for the potential clinical application of 1,25(OH)_2_D_3_ in overcoming drug resistance during liver cancer therapy.

## Figures and Tables

**Table 1:** Binding energies of 1,25(OH)_2_D_3_ and FOXO3A.

Compound	Key Target	PDB ID	Binding Energy (−kcal/mol)
1,25(OH)_2_D_3_	FOXO3A	2UZK	−8.62
Maxacalcitol	FOXO3A	2UZK	−8.47
Calcipotriol	FOXO3A	2UZK	−7.99
Seocalcitol	FOXO3A	2UZK	−8.54
Tacalcitol	FOXO3A	2UZK	−8.57

**Table 2:** q-values of 1,25(OH)_2_D_3_ in combination with different concentrations of sorafenib.

Group	Joint Concentration	q-Value
Sorafenib + 1,25(OH)_2_D_3_	4.3 + 5	2.496671581
Sorafenib + 1,25(OH)_2_D_3_	8.6 + 5	1.719277168
Sorafenib + 1,25(OH)_2_D_3_	17.2 + 5	1.401718285
Sorafenib + 1,25(OH)_2_D_3_	34.4 + 5	1.099641231
Sorafenib + 1,25(OH)_2_D_3_	68.8 + 5	1.015836282

## Data Availability

Data will be made available on request.

## Supplementary Materials

The following supporting information can be downloaded at https://www.mdpi.com/article/10.3390/cimb47050319/s1.
