# Supplementary material for: Exploring the Molecular Mechanism of 1,25(OH)2D3 Reversal of Sorafenib Resistance in Hepatocellular Carcinoma Based on Network Pharmacology and Experimental Validation
Source: Curr Issues Mol Biol. 2025 Apr 29;47(5):319. doi: 10.3390/cimb47050319 (PMC12109729; doi:10.3390/cimb47050319)
Supplement: Supplementary file 1 [file cimb-47-00319-s001.zip › Figure S1, Tables S1 and S2 legends.pdf]

Figure S1. The IC50 value of 1,25(OH)2D3.
Tables S2. Predicted targets of 1,25(OH)2D3 and its derivatives.
Tables S3. Differentially expressed genes of sorafenib resistance in liver cancer.
I have provided supplementary explanations in the supplementary materials.
