# Supplementary material for: Exploring the Molecular Mechanism of 1,25(OH)2D3 Reversal of Sorafenib Resistance in Hepatocellular Carcinoma Based on Network Pharmacology and Experimental Validation
Source: Curr Issues Mol Biol. 2025 Apr 29;47(5):319. doi: 10.3390/cimb47050319 (PMC12109729; doi:10.3390/cimb47050319)
Supplement: Supplementary file 1 [file cimb-47-00319-s001.zip › Table S1.pdf]

Table 1. the chemical structure of 1,25(OH)2D3 and its derivatives

Number	Compound name	Compound structure
1	calcitriol	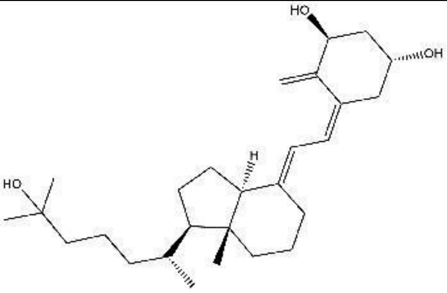
2	Maxacalcitol	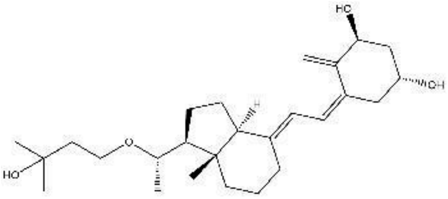
3	Seocalcitol	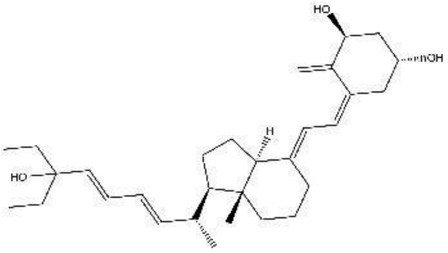
4	Calcipotriol	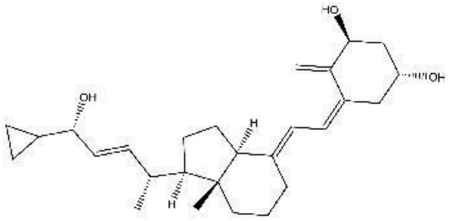
5	Tacalcitol	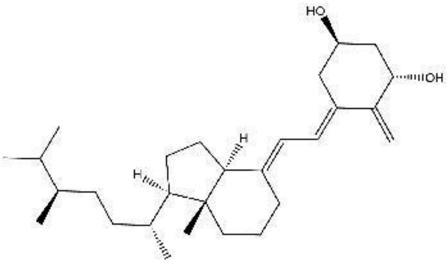
